# Norovirus Epidemiology and Genetic Diversity in Leipzig, Germany during 2013–2017

**DOI:** 10.3390/v13101961

**Published:** 2021-09-29

**Authors:** Nora Ennuschat, Sabine Härtel, Corinna Pietsch, Uwe G. Liebert

**Affiliations:** Institute of Virology, Leipzig University, 04103 Leipzig, Germany; nora.ennuschat@protonmail.com (N.E.); sabine.haertel@gmx.net (S.H.)

**Keywords:** viral gastroenteritis, molecular epidemiology, genotyping, diarrhea, viral diversity, anti-norovirus vaccines

## Abstract

Globally and in all age groups, noroviruses are a main cause of gastroenteritis. To assess their local epidemiology and genetic diversity, stool samples of 7509 inpatients with gastrointestinal complaints from all age groups were analyzed. After detection of norovirus genogroup I and II RNA by real-time RT-PCR, viral capsids were genotyped by partial nucleic acid sequencing. In the case of GII.2 strains, polymerase genotypes were also assessed. Between October 2013 and September 2017, presence of norovirus RNA was shown in 611 samples (8.1%), of which 610 (99.8%) were typed successfully. Norovirus positivity rate was higher in patients aged below five years (14.8%) than in older patients (5.7%). Among the 611 norovirus positive samples, GII.4 (56.6%) strains prevailed, followed by GII.6 (11.3%), GII.3 (11.0%) and GII.2 (9.5%). The most common genogroup I (GGI) genotype was GI.3 (3.6%). In addition, rare genotypes such as GII.13, GII.14 and GII.26 were detected. Interestingly, GII.3 infections were most common in children under the age of five years. Assessment of polymerase genotypes in GII.2 viruses showed a shift from P2 to P16, with higher diversity in P2 sequences. The varying distribution of norovirus genotypes depending on season, age and setting of infection highlights the importance of frequent genotyping as a basis for vaccine development and needful adjustments.

## 1. Introduction

Globally, noroviruses are estimated to cause about 18% of all cases with acute gastroenteritis in patients of all age groups [1,2]. Following the introduction of vaccination against rotavirus, human noroviruses are the main cause for viral gastroenteritis in children [3,4,5]. Noroviruses are known to have a seasonality, with peaks during the winter months [6], and are mainly transmitted via the fecal-oral route or contaminated food [7,8]. Both community-acquired and nosocomial infections are common and may result in outbreaks [9]. The incubation period lasts from 18 to 48 h [10]. The main symptoms of norovirus infections are vomiting and diarrhea, which are usually self-limiting and of a short duration of one to three days [11]. Though especially the elderly, immunocompromised and children younger than five years can suffer from severe or prolonged illness [12,13,14]. 

The 7.5 kb genome of human noroviruses consists of three open reading frames (ORFs) [15]. Thereof, ORF1 encodes for at least six non-structural proteins, including the RNA-dependent RNA-polymerase (RdRp). The structural proteins, viral protein 1 (VP1, capsid) and 2 (VP2), are encoded in ORF2 and 3, respectively [16]. Based on partial ORF1 and ORF2 sequences, noroviruses can be classified into at least 10 genogroups. Within the human pathogenic genogroups GI, GII, GIV, GVIII and GIX, at least 35 different human pathogenic genotypes, 9 in GI, 23 in GII and one each in GIV, GVIII and GIX, have been described, respectively [17]. Within the human pathogenic genogroups (GG), GGII and GGI viruses are most common. Both can be assessed in stool samples by real-time RT-PCR using genogroup-specific primer sets targeting a conserved section of the genome located at the ORF1/ORF2 junction [18]. As recombination events may occur during norovirus co-infections, dual-typing, i.e., genotyping of the viral capsid and the polymerase gene, is increasingly recommended [17].

Especially in young children, norovirus incidence and hospitalization rates are high, making them a possibly efficient target group for vaccination [19]. Currently, clinical trials for anti-norovirus vaccines are still ongoing [20,21,22]. Attempts for vaccine development are being made, however due to the high diversity of noroviruses and the rapid antigenic drift in common genotypes [23,24] it remains challenging [25,26]. Presumably, multivalent vaccines with adjustment now and then are needed [19]. Consequently, studies on the genetic diversity, evolution and variation of noroviruses over time, age and setting are essential [19].

Thus, the local epidemiology and genetic diversity of human noroviruses was assessed in inpatients at a tertiary medical center in Leipzig, Germany during four subsequent seasons with a special focus on children under five years of age, as well as on a potential upsurge of specific viral strains or genotypes. 

## 2. Materials and Methods

### 2.1. Study Population

Between October 2013 and September 2017, 7509 stool samples collected from inpatients of all age groups at Leipzig University Hospital who suffered from vomiting, acute or chronic diarrhea or other gastrointestinal symptoms, such as abdominal pain or discomfort, were included in the study. To avoid any bias created by persistent norovirus infections, no follow-up samples, i.e., samples within 28 days after initial testing, were included.

### 2.2. RNA Extraction, Detection, Sequencing and Typing

Specimens were diluted with phosphate buffered saline to a 10% suspension. Total RNA was extracted using NucliSens easyMAG system (bioMérieux, Marcy l’Etoile, France) and stored at −80 °C. Real-time RT-PCR was performed to detect norovirus RNA and to assess the viral genogroup, GGI and GGII, respectively [27,28,29]. Information on all primers and probes used in this study can be found in the Supplementary Material, Table S1. Amplicons were detected optically using fluorescent nucleic acid probes in glass capillaries (Light cycler 2.0, Roche, Mannheim, Germany).

For capsid genotyping of all GII and GI noroviruses, partial capsid genomes were amplified by RT-PCR using different primer sets (Table S1). If amplification with primers NV107c(s) [30] and NV156(as) [31] failed, alternative reverse primers NV300II [31], G2SKR [32] and G2R1 [32] were used in GGII strains. For analysis of genetic diversity in GII.2 samples, the partial viral RdRp gene and almost the complete VP1 gene was assessed using GII.2 specific primers [33,34,35,36] (Table S1) in RT-PCR and subsequent nucleic acid sequencing. 

Amplicons were analyzed using agarose gel electrophoresis. Thereafter, gel-purified amplicons (PCR Clean-Up System, Promega, Madison, WI, USA) were sequenced (Big Dye Terminator v1.1 Cycle Sequencing Kit and ABI 3500 Genetic Analyzer, PE Applied Biosytems, Foster City, CA, USA). All obtained sequences were submitted to GenBank (Accession numbers MZ702937 to MZ702975, as well as MZ708031 to MZ708604 and MF352143).

### 2.3. Analysis of Sequences and Phylogeny

Sequence electropherograms were analyzed and adjusted using Geneious software v6.06 (Biomatters Ltd., Auckland, New Zealand) and genotypes were assigned using the publicly available Norovirus Typing Tool (https://www.rivm.nl/mpf/typingtool/norovirus (accessed on 13 September 2021) [37]. GII.4 variants were assigned by the Norovirus Typing Tool, and in the case of missing assignments, subsequent Human Calicivirus Typing Tool HuCaT (https://norovirus.ng.philab.cdc.gov/bctyping.html accessed on 13 September 2021) analysis. 

Phylogenetic analysis of GII.2 ORF1 (409 to 1003 nt referring to GenBank accession number X81879), GII.2 ORF2 (983 to 2569 nt referring to GenBank accession number X81879), and partial GII.3 (851 to 1411 nt referring to GenBank accession number U02030) sequences was performed using maximum likelihood algorithm with 1000 bootstrap replicates in MEGA 5. By the same approach, partial GII.4 (1411 to 1675 nt referring to GenBank accession number X76716) were analyzed for topology tree building. Additionally, pairwise distances were calculated using the Jukes Cantor model to analyze genetic divergence in GII.2, GII.3 and GII.4 samples.

### 2.4. Classification of Nosocomial and Community-Acquired Infections 

For each patient, the infection was classified as community-acquired or nosocomial based on symptom onset, admission date, sampling date and individual medical chart reviews. Community-acquired was defined as symptoms occurring before or within 48 h after admission and nosocomial was defined as symptoms occurring more than 48 h after admission [38]. Twenty patients living in long-term care facilities were included as nosocomial infections because of their special living conditions [39].

### 2.5. Statistical Analysis

Statistical analysis was carried out using IBM SPSS Statistics version 25 (IBM, Armonk, NY, USA). The dataset was analyzed using binary logistic regression. The association of norovirus infection (“yes” and “no”) and sex, age, season and time of testing (defined as month of the year) was analyzed. Within the dataset of all norovirus-positive samples, the association of community-acquired infection (“yes” and “no”) and sex, age and genotype were analyzed using binary logistic regression as well.

Odds ratios (ORs) were determined by using four-fold tables.

Kruskal-Wallis test was used to determine whether pairwise distances in GII.2 RdRp sequences were significantly different in the three groups.

*p*-values of <0.05 were considered statistically significant. 

### 2.6. Ethical Clearance

The study was approved by the Ethics Committee of Leipzig University (26 September 2016, AZ 298/16-ek).

## 3. Results

Norovirus RNA was detected in 611 out of 7509 (8.1%) samples. There were no significant differences regarding patients’ gender (*p* > 0.2) and season (*p* > 0.2). However, the analysis showed significant differences in age (*p* < 0.01) and month of infection (*p* < 0.01) (Table 1).

The median age (range) of all 7509 patients with gastrointestinal complaints was 49 years (0,99 years) compared to five years (0,93 years) in the norovirus-positive group. Children aged younger than five years had a higher risk for testing positive for noroviruses compared to older patients with an OR = 2.847 (95%CI 2.407, 3.367). The risk for being tested positive for noroviruses was highest between October and March (OR = 3.826; 95%CI 3.110; 4.705) (Table 1). 

Altogether, 316 (51.7%) of the norovirus-positive samples were classified as community-acquired and 295 (48.3%) as nosocomial. 

**Figure 1:**
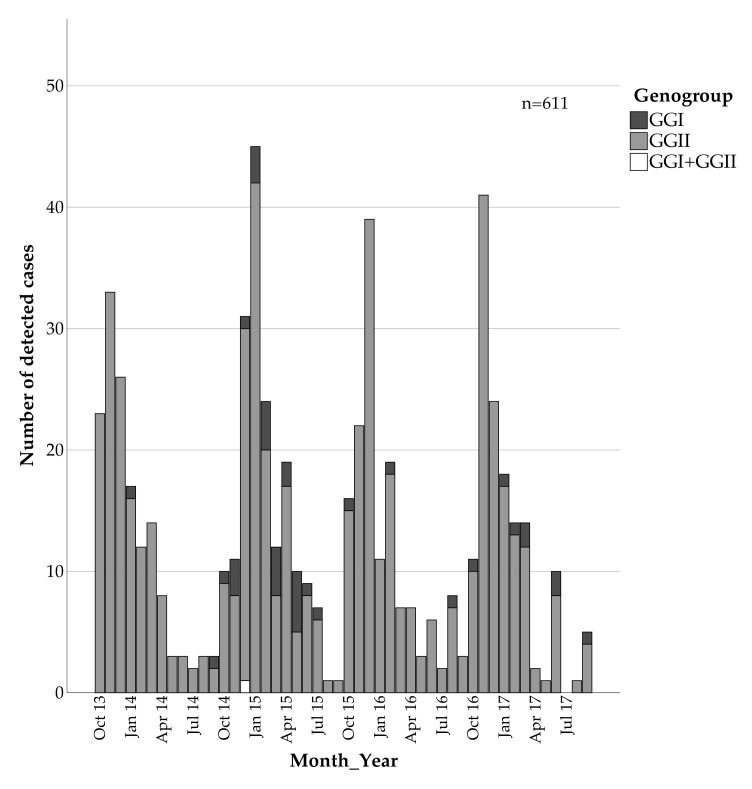
Monthly distribution of detected norovirus GGI and GGII strains, Leipzig University Hospital, October 2013–September 2017.

Genotyping using partial capsid sequences was successful in 610 of 611 samples (99.8%). The majority of the norovirus-positive samples were classified as GGII (93.6%) while only 38 samples were of GGI (6.2%) and one sample contained both GGI and GGII viral RNA (0.16%) (Figure 1). The risk for GGI infections was significantly higher among community-acquired compared to nosocomial infections, with an OR = 3.234 (95%CI 1.504, 6.953) (Table 2).

Among GGI, the most frequently appearing genotype was GI.3 (3.6%), followed by GI.2 (1.1%). Other GGI genotypes identified were GI.1 (0.2%), GI.4 (0.2%), GI.5 (0.3%), GI.6 (0.8%) and one mixed GI.3 and GII.4 infection (0.2%).

**Figure 2:**
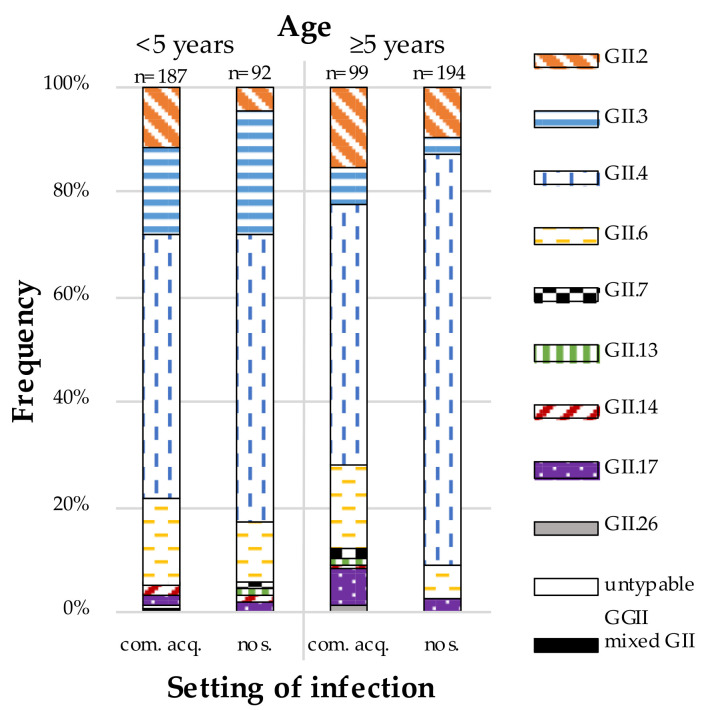
Distribution of GII norovirus genotypes according to age and mode of acquisition, Leipzig University Hospital October 2013–September 2017; com. acq. stands for community-acquired infections and nos. stands for nosocomial infections.

More than half of the norovirus-positive samples were typed as GII.4 (56.6%), with GII.6 (11.3%) being the second most common genotype, followed by GII.3 (11.0%) and GII.2 (9.5%). In contrast, GII.7 (0.7%), GII.13 (0.3%), GII.14 (0.8%), GII.17 (2.9%) and GII.26 (0.2%) were rarely detected. One sample contained a mixed infection of GII.2 and GII.4 (0.2%) noroviruses and one sample remained untypable (0.2%) (Figure 2). 

Binary logistic regression, performed in the dataset of norovirus-positive samples, regarding the setting of infection (community-acquired vs nosocomial) resulted in significant results for age (*p* < 0.01) and genotype (*p* < 0.01) (Table 2). In children younger than five years, 67.0% of GII samples were classified as community-acquired compared to 33.8% of GII samples in patients aged five years and older (Figure 2). According to the data, children younger than five years have a significant lower risk to be nosocomially infected with noroviruses, with OR = 0.268 (95%CI 0.191, 0.374) (Table 2). 

Altogether, GII.4 was more frequent in patients with nosocomial infections (OR = 2.579; 95%CI 1.853, 3.591) while GII.6 (OR = 2.022; 95%CI 1.193, 3.429) was more frequent in community-acquired infections (Table 2). Concurrently, GII.4 noroviruses seemed to be more common among patients older than five years and GII.6 infections among patients younger than five years, but their different distribution in the two age groups turned out not to be significant after stratification by the setting of infection (community-acquired vs nosocomial). In contrast, GII.3 noroviruses remained significantly more common in patients younger than five years (OR 4.744; 95%CI 2.570, 8.755) even after the stratification.

The norovirus genotype distribution differed between the seasons (Table 3). In children younger than five years, the data showed more GII.4 (53 of 88 samples) and GII.6 (24 of 88 samples) infections in season 2013/2014 compared to the following seasons. The risk of a GII.4 (OR = 1.808; 95%CI 1.091, 2.996) or GII.6 infection (OR = 4.083; 95%CI 2.064, 8.079) was significantly higher in season 2013/2014. In 2014/2015, the risk of a GII.3 infection was significantly higher (OR = 3.091; 95%CI 1.638, 5.834) with 22 of 60 samples being typed as GII.3. GII.2 was more frequent in season 2015/2016 (14 of 87 samples; OR = 3.292; 95%CI 1.431, 7.576) as well as in season 2016/2017 (9 of 44 samples), with OR = 3.220 (95%CI 1.332, 7.787) (Table 3).

**Figure 3:**
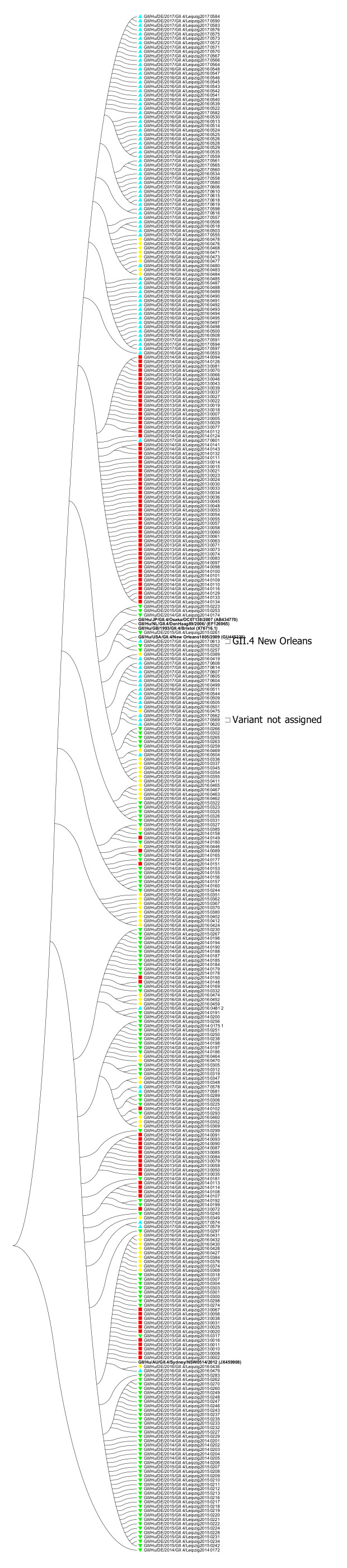
Phylogenetic analysis of norovirus GII.4 genotypes based on Maximum Likelihood estimations (1000 bootstraps) of partial ORF2 nucleic acid sequences. Only topology is shown, ignoring the branch lengths. Red squares indicate sequences of season 2013/2014, green arrow heads facing downwards indicate sequences of season 2014/2015, yellow diamonds indicate sequences of season 2015/2016 and blue arrow heads facing upwards indicate sequences of season 2016/2016. Labels in bold indicate reference strains, with GenBank accession numbers shown in parenthesis. All sequences without labeled variants are GII.4 Sydney strains.

In 348 samples partial GII.4 sequences were obtained, of which two samples contained a mixed infection with GII.2 and GI.3, respectively. Within GII.4 samples, the predominant variant was GII.4 Sydney (99.4%) while GII.4 New Orleans was assigned in one sample (0.3%) and in one sample the variant could not be assigned (0.3%) (Figure 3 and as a high-resolution PDF Figure S1).

**Figure 4:**
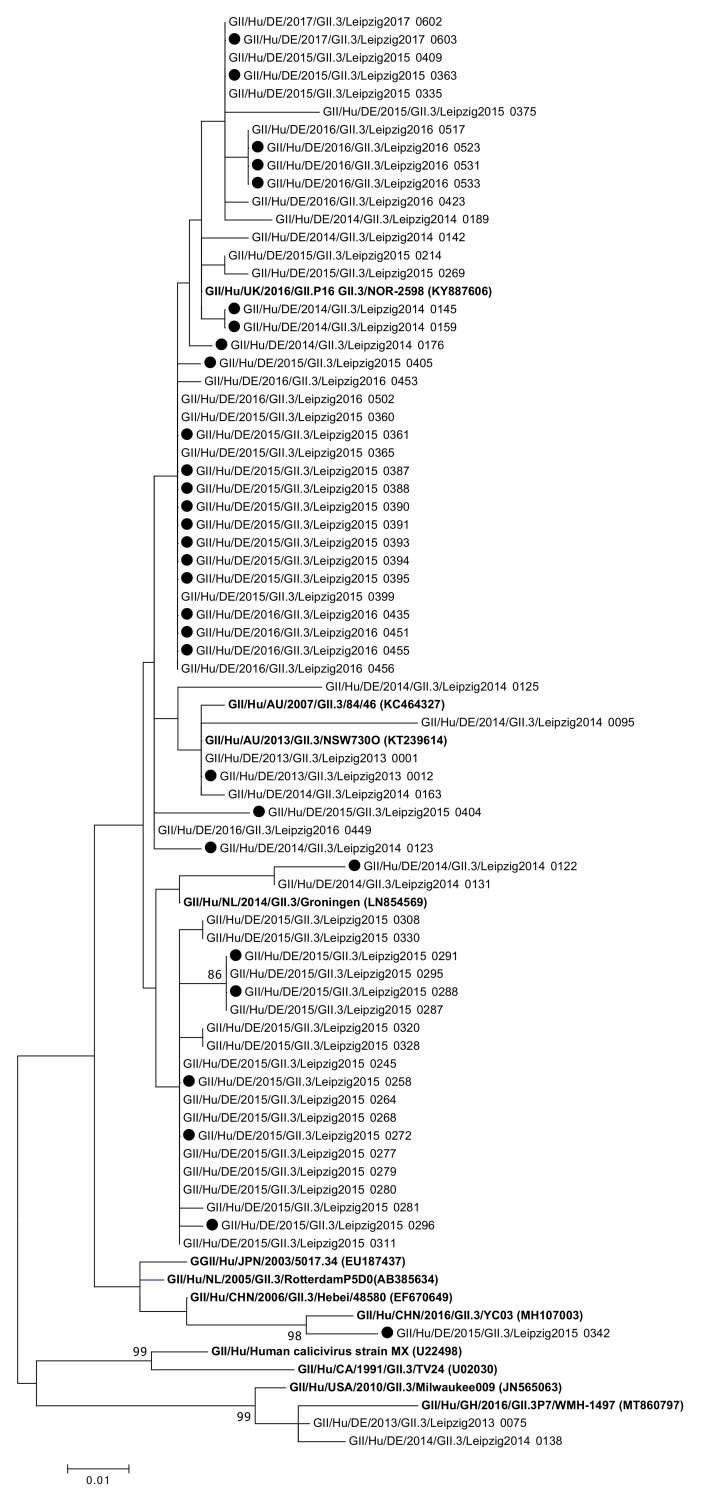
Phylogenetic analysis of norovirus GII.3 genotypes based on Maximum Likelihood estimations of partial ORF2 nucleic acid sequences. Exclusively, bootstrap values (1000 replicates) above 80% are shown. Black circles indicate sequences of nosocomial infections. Labels in bold indicate reference strains, with GenBank accession numbers shown in parenthesis.

GII.3 sequences were detected and analyzed in 69 samples (Figure 4).

**Figure 5:**
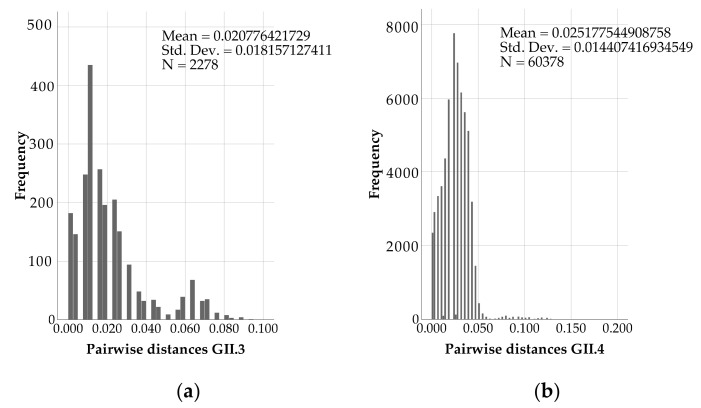
Pairwise distances within norovirus (**a**) GII.3 and (**b**) GII.4 sequences calculated by Jukes Cantor method in MEGA.

Between the obtained partial GII.3 sequences (analyzed length 263 bp), the pairwise distance was 0.0208 ± 0.0182 (mean ± SD) (Figure 5a). The pairwise distance value within GII.4 sequences (analyzed length 264 bp) was 0.0252 ± 0.0144 (mean ± SD) (Figure 5b). The difference in mean pairwise distances tested to be significant by the Mann-Whitney test (*p* < 0.001).

Capsid genotype GII.2 was shown by partial ORF2 sequencing in 60 samples. Therefore, partial ORF1 sequences were obtained in 58 samples (96.7%) and almost complete ORF2 sequences in 57 samples (95.0%).

**Figure 6:**
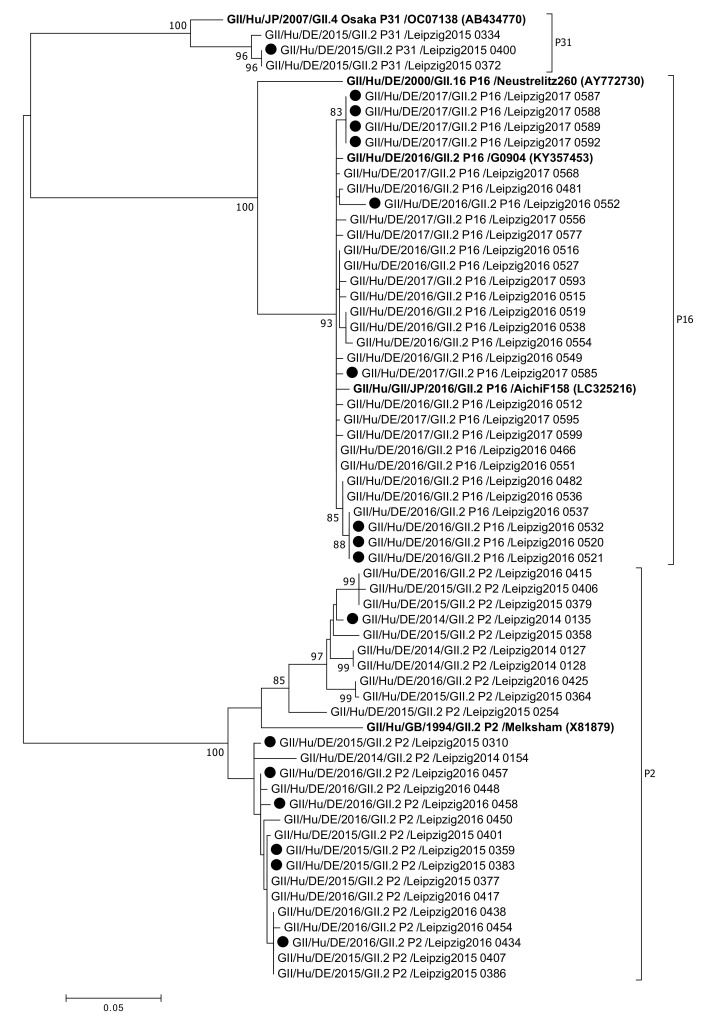
Phylogenetic analysis of norovirus GII.2 polymerase genotypes based on Maximum Likelihood estimations of partial ORF1 nucleic acid sequences. Exclusively, bootstrap values (1000 replicates) above 80% are shown. Black circles indicate sequences of nosocomial infections. Labels in bold indicate reference strains with GenBank accession numbers shown in parenthesis.

Three different polymerase types were shown: P16 (29 samples), P2 (26 samples) and P31 (3 samples) (Figure 6).

**Figure 7:**
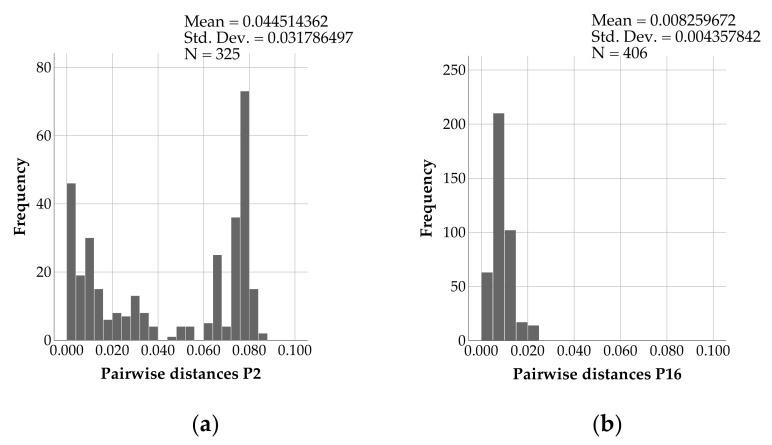
Pairwise distances within norovirus (**a**) GII.2[P2] and (**b**) GII.2[P16] sequences calculated by Jukes Cantor method in MEGA.

While mainly genotype GII.2[P2] strains were detected up to season 2015/2016, from July 2016 onwards only GII.2[P16] was shown. Between all the obtained partial ORF1 sequences of GII.2 samples (length 594 bp), the pairwise distance was 0.1474 ± 0.1107 (mean ± SD). The pairwise distance value within P2 sequences was 0.0445 ± 0.0318 (mean ± SD), whereas in P16 sequences it was 0.0083 ± 0.0044 (mean ± SD) (Figure 7) and in P31 genotypes 0.0068 ± 0.0059 (mean ± SD). According to the mean pairwise distances, less genetic diversity was found within the sequences of P16 strains compared to sequences of P2 strains. The differences in pairwise distances in the three groups tested significant by the Kruskal-Wallis test.

## 4. Discussion

This study revealed a high genetic diversity of human norovirus strains in inpatients in Leipzig, Germany from October 2013 to September 2017. Consistent with other studies, norovirus genotypes varied according to season and patient’s age [40,41,42]. The study underlines the high prevalence of GII.4, regardless of age or setting of the infection, thus verifying GII.4 as a promising vaccination candidate [25]. The proportion of GII.4 noroviruses was especially high in nosocomial infections. In general, a higher variety of genotypes was found in community-acquired than in nosocomial infections. However, the distribution of norovirus genotypes circulating in the community may be different, as only patients seeking inpatient treatment at Leipzig University Hospital were included in this study. Thus, if certain norovirus genotypes are associated with milder disease outcomes, these may be underrepresented in the present study.

A strength of this study is the large number of analyzed samples compared to similar studies [43,44,45]. Another one is the possibility of a direct comparison of diversity in the two age groups of patients below five years of age versus older patients at the same site and during the same period. 

Besides adults with a higher risk for severe or prolonged illness (e.g., immunocompromised patients) [14], children may be a possibly efficient target group for vaccination. Therefore, genotype distribution in this age group is of special interest. Our study identified GII.3, GII.6 and GII.2 as common in children younger than five years in Leipzig, Germany. Not only in Germany but also in other areas such as the USA [46], Japan [43] and India [44], GII.3 is especially common among children [47]. Nucleotide variation among GII.3 are described to be lower compared to GII.4 [23,46], confirmed by a significant difference in pairwise distances of GII.3 and GII.4 sequences analyzed in this study. It is hypothesized that this may be due to different characteristics of the infected subpopulations. While GII.3 infects a constantly renewed pool of young children, GII.4 repeatedly infects adults, escaping the patient’s immune response due to higher evolution rates [46]. Limited evolution in GII.3 epitopes as well as cross-reactivity of antibodies among GII.3 strains was described [48], adding to being an interesting vaccine candidate.

Ideally, vaccination would not only protect patients against the most frequent genotype GII.4 noroviruses, but also induce protection against other GGII noroviruses; according to the present data, especially against GII.3, GII.6 and GII.2 strains.

The present study showed a higher risk of children under five years to test positive for noroviruses. Reasons for this may not only be higher infection rates among children, but also a higher possibility of seeking medical care or suffering from severe disease, as well as insufficient hand hygiene in this age group [49,50]. Adults suffering from mild gastrointestinal symptoms may not have sought treatment at Leipzig University Hospital, possibly leading to a bias in patients’ age. Additionally, gastroenteritis might be the primary diagnosis in children, while adults seek medical care for other reasons (e.g., chronic diseases) and get tested for noroviruses whenever they suffer from gastrointestinal complaints [51].

Genogroup I norovirus infections being more frequently community-acquired than nosocomial is consistent with the fact that GGI noroviruses are more likely to be foodborne than person-borne [52].

As there is no vaccination available yet, a possible way to reduce the burden of norovirus infections may be the reduction of nosocomial infections. The classification of infections as nosocomial or community-acquired used in this study may be vague because of the short incubation period of 18 to 48 h [10]. Consistent with the World Health Organization (WHO) and other studies on norovirus infections, we defined community-acquired infections as symptoms being present at admission or occurring within 48 h afterwards. Some studies classified infections five days after admission as nosocomial and samples between 48 h and 5 days as indeterminate [38,45,51,53]. Due to the short incubation period of noroviruses, we used the stricter definition of every infection occurring more than 48 h after admission as being nosocomial, which is in line with the WHO’s definition and comparable to another study from Germany [51,53]. The distribution of community-acquired (51.7%) and nosocomial (48.3%) infections in our study was similar to results obtained in a study covering all of Germany [51]. A study carried out in Denmark showed a percentage of 63% nosocomial infections [38]. A possible reason for this may be the higher percentage of older patients in their study. Still, their definition of nosocomial infections was less strict (symptoms starting five days after admission) and would lead to a smaller percentage of nosocomial infections than our definition, so there may be a real difference. 

In GII.2 noroviruses, a more detailed analysis of both ORF1 and ORF2 was carried out due to a rise in norovirus infections with GII.2 [P16] during the season 2016/2017 in Germany [40]. Generally, evolutionary rates of noroviruses seem to differ between ORF1 and ORF2, as shown for GII.4 and GII.3 noroviruses, with lower rates in ORF1 [54]. Among the GII.2 sequences obtained in this study, a significantly higher genetic diversity was found in P2 sequences compared to P16 sequences. This may indicate a shorter circulation period of GII.2[P16] sequences in the population studied and is consistent with GII.2[P16] sequences not occurring before July 2016 in the time covered. Furthermore, the increase of GII.2[P16] infections in 2016/2017 is in line with another publication covering Germany [40]. 

Assessing the polymerase genotype in all detected noroviruses would possibly give further insights into the molecular diversity of circulating noroviruses. While not being implemented in the present study’s approach, recently established integrated dual-genotyping protocols for noroviruses will possibly further improve future studies and therefore our knowledge on this heterogeneous human pathogenic virus [17].

## 5. Conclusions

Overall, the data show a great diversity of noroviruses detected with some genotypes being predominant, making them possibly interesting vaccine candidates. The findings are in line with similar studies, characterizing GII.4 as the overall most present genotype, but also showing differences in occurring genotypes regarding age and the setting of the infection. Generally, children under five years of age are less prone to nosocomial infection, and GII.3 as well as GII.6 were especially frequent in this cohort. The observed upsurge of norovirus GII.2 infections by introduction of GII.2[P16] viruses document the ever-changing nature of norovirus epidemiology and diversity. Accordingly, norovirus genotype surveillance needs to be ongoing to provide a meaningful basis for targeted vaccine development. 

## Figures and Tables

**Table 1:** Analysis of Characteristics of 7509 Patients with Gastrointestinal Complaints.

Characteristics	Norovirus Positivity		OR (95%CI)	*p*
	Yes	No		
**Sex**			0.909 (0.770, 1.073)	>0.2
Male	333	3596		
Female	278	3302		
**Season**			NA	>0.2
2013/2014	147	1722		
2014/2015	180	1780		
2015/2016	143	1660		
2016/2017	141	1736		
**Age**			2.847 (2.407, 3.367)	<0.001
<5 years	294	1695		
≥5 years	317	5203		
**Time of** **infection**			3.826 (3.110; 4.705)	<0.001
October to March	494	3619		
April to September	117	3279		

OR: Odds ratio; CI: confidence interval; *p*: *p*-value; NA: not applicable. For statistical analysis patients are stratified by sex, season, age and time of infection (left column, bold).

**Table 2:** Analysis of Characteristics of Norovirus-Positive Samples.

Characteristics	Setting of Infection		OR (95%CI)	*p*
	Community Acquired	Nosocomial		
**Genotype**			NA	<0.001
GI.1	1	0		
GI.2	5	2		
GI.3	17	5		
GI.4	1	0		
GI.5	2	0		
GI.6	3	2		
GII.2	36	22		
GII.3	38	29		
GII.4	144	202		
GII.6	46	23		
GII.7	3	1		
GII.13	1	1		
GII.14	4	1		
GII.17	11	7		
GII.26	1	0		
GI.3 and GII.4	1	0		
GII.2 and GII.4	1	0		
**Genogroup**			3.234 (1.504, 6.953)	0.002
GGI	29	9		
GGII	285	286		
**Age**			0.268 (0.191, 0.374)	<0.001
<5 years	201	94		
≥5 years	115	201		
**GII.3**			0.795 (0.476, 1.326)	>0.4
Yes	38	29		
No	277	266		
**GII.4**			2.579 (1.853, 3.591)	<0.001
Yes	144	202		
No	171	93		
**GII.6**			2.022 (1.193, 3.429)	0.01
Yes	46	23		
No	269	272		

OR: Odds ratio; CI: confidence interval; *p*: *p*-value; NA: not applicable. For statistical analysis samples and patients are stratified by genotype, genogroup and patients’ age, respectively (left column, bold).

**Table 3:** Norovirus GII genotypes by season in children < 5 years, Leipzig University Hospital, October 2013–September 2017.

Norovirus Genotype	2013/2014	2014/2015	2015/2016	2016/2017	Σ
GII.2	1	1	14	9	25
GII.3	9	22	19	3	53
GII.4	53	30	34	28	145
GII.6	24	6	9	2	41
GII.17	1	0	5	0	6
Other GGII	0	0	6	2	8
Untypable GGII	0	1	0	0	1
**Σ**	88	60	87	44	279

## Data Availability

Identified sequences were submitted to GenBank (accession No. MZ702937 to MZ702975, as well as MZ708031 to MZ708604 and MF352143).

## Supplementary Materials

The following are available online at https://www.mdpi.com/article/10.3390/v13101961/s1, Table S1: Primers and probes; Figure S1: Phylogenetic analysis of norovirus GII.4 genotypes based on Maximum Likelihood estimations (1000 bootstraps) of partial ORF2 nucleic acid sequences.
