# Supplementary material for: Norovirus Epidemiology and Genetic Diversity in Leipzig, Germany during 2013–2017
Source: Viruses. 2021 Sep 29;13(10):1961. doi: 10.3390/v13101961 (PMC8541062; doi:10.3390/v13101961)
Supplement: Supplementary file 1 [file viruses-13-01961-s001.zip › Figure S1.pdf]

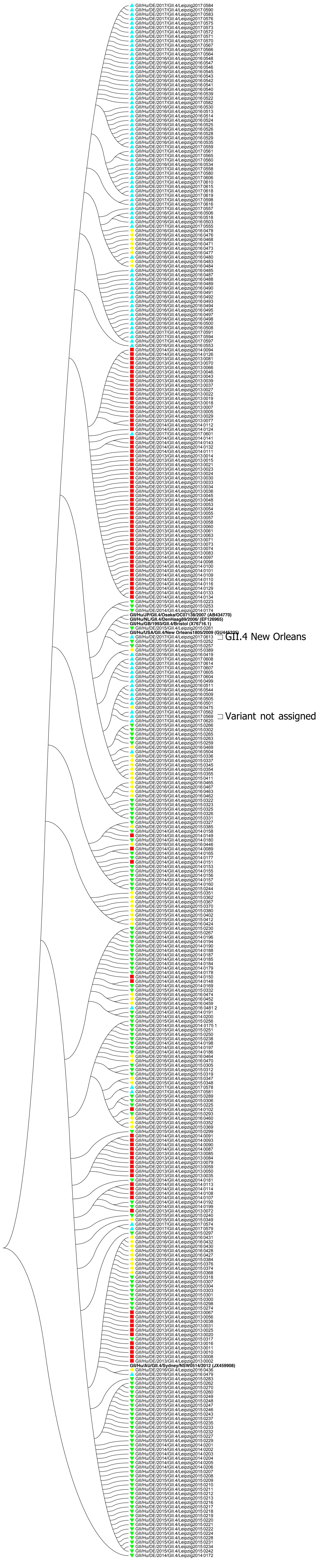

GII.4 New Orleans
Variant not assigned

Figure S1: Phylogenetic analysis of norovirus GII.4 genotypes based on Maximum Likelihood estimations (1000 bootstraps) of partial ORF2 nucleic acid sequences. Only topology is shown, ignoring the branch lengths. Red squares indicate sequences of season 2013/2014, green arrow heads facing downwards indicate sequences of season 2014/2015, yellow diamonds indicate sequences of season 2015/2016 and blue arrow heads facing upwards indicate sequences of season 2016/2016. Labels in bold indicate reference strains, with GenBank accession numbers shown in parenthesis. All sequences without labeled variants are GII.4 Sydney strains.
