# Supplementary material for: Norovirus Epidemiology and Genetic Diversity in Leipzig, Germany during 2013–2017
Source: Viruses. 2021 Sep 29;13(10):1961. doi: 10.3390/v13101961 (PMC8541062; doi:10.3390/v13101961)
Supplement: Supplementary file 1 [file viruses-13-01961-s001.zip › Table S1.pdf]

Supplementary Material

Table S1: Primers and probes

Genogroup/ genotype	Name	Amplicon size (bp)	Direction	Position	Sequence 5'→ 3')	Reference
**Norovirus real-time RT-PCR**						
GI	NV GI 192(s)	98	F	5282-5300[a]	GCYATGTTCCGCTGGATGC	[1]
	NV GI 193(as)		R	5379-5359[a]	CGTCCTTAGACGCCATCATCA	[1]
	TM9-P		F	5321-5335[a]	TGGACAGGAGATCGC	[1]
GII	COG2Fwobble	98	F	5003-5028[b]	CARGARBCNATGTTYAGRTGGATGA	[2]
	COG2R		R	5100-5080[b]	TCGACGCCATCTTCATTCACA	[2]
	RING2-TP		F	5050–5069[b]	FAM-TGGGAGGGCGATCGCAATCT-	[2]
**Norovirus genotyping VP1**						
GI	NV192(s)	390	F	5282-5300[a]	GCYATGTTCCGCTGGATGC	[1]
	NV285(as)		R	5671-5652[a]	CCAACCCARCCATTRTACAT	[3]
GII	NV107c (s)	381	F	5007-5026[b]	AICCIATGTTYAGITGGATG	[1]
	NV156 (as)		R	5387-5367[b]	ACCKGCATAACCATTRTACAT	[3]
GII	NV107c (s)	372	F	5007-5026[b]	AICCIATGTTYAGITGGATG	[1]
	NV300/II (as)		R	5378-5359[b]	CYAGGKGCYTGIACAAARTT	[3]
GII	NV107c (s)	383	F	5007-5026[b]	AICCIATGTTYAGITGGATG	[1]
	G2SKR (as)		R	5389-5367[b]	CCRCCNGCATRHCCRTTRTACAT	[4]
GII	NV107c (s)	378	F	5007-5026[b]	AICCIATGTTYAGITGGATG	[1]
	G2R1 (as)		R	5384-5364[b]	TGCATAACCATTRTACATTCT	[4]
**Norovirus GII.2 genotyping**						
GII.2 RdRp	ORF1-8Fwobble	611	F	4489-4508[b]	CCAATGGAATTCCATCKCMC	Modified [5]
	COG2R		R	5100-5080[b]	TCGACGCCATCTTCATTCACA	[6]
	ORF1-7F	720	F	3824-3842[b]	GGC TGC CAA GAA AAC CAT C	[7]
	ORF1-7R		R	4543-4524[b]	ACCTCAGAAAGTGCACAGAG	[7]
	ORF1-8F	610	F	4490-4509[b]	CCAATGGAATTCCATCGCCC	[7]
	ORF1-8R		R	5099-5080[b]	CGACGCCATCTTCATTCACA	[7]
GII.2 VP1	NV-GII.2_VP1_1 (s)	862	F	5073-5093[c]	GAATGAAGATGGCGTCGAATG	[8]
	NV-GII.2_VP1_1 (as)		R	5934-5912[c]	TTRAAWGCRCAAATRCCACTRAC	[8]
	NV-GII.2_VP1_2 (s)	909	F	5786-5806[c]	TCYAATTCHAGRTTYCCAGTG	[8]
	NV-GII.2_VP1_2 (as)		R	6694-6678[c]	YCTTCTRCGCCCATTYC	[8]

Position refers to GenBank accession numbers [a] M87661(NoV GI, Norwalk); [b] X86557 (NoV GII, Lordsdale); [c] LC145802 NoV GII.2, Osaka)

Supplement References:

1. Hoehne, M.; Schreier, E. Detection of Norovirus Genogroup I and II by Multiplex Real-Time RT- PCR Using a 3'-Minor Groove Binder-DNA Probe. *BMC Infectious Diseases* **2006**, *6*, doi:10.1186/1471-2334-6-69.
2. Kageyama, T.; Kojima, S.; Shinohara, M.; Uchida, K.; Fukushi, S.; Hoshino, F.B.; Takeda, N.; Katayama, K. Broadly Reactive and Highly Sensitive Assay for Norwalk-Like Viruses Based on Real-Time Quantitative Reverse Transcription-PCR. *Journal of Clinical Microbiology* **2003**, *41*, 1548–1557, doi:10.1128/JCM.41.4.1548-1557.2003.
3. Bernard, H.; Höhne, M.; Niendorf, S.; Altmann, D.; Stark, K. Epidemiology of Norovirus Gastroenteritis in Germany 2001–2009: Eight Seasons of Routine Surveillance. *Epidemiol. Infect.* **2014**, *142*, 63–74, doi:10.1017/S0950268813000435.
4. Kojima, S.; Kageyama, T.; Fukushi, S.; Hoshino, F.B.; Shinohara, M.; Uchida, K.; Natori, K.; Takeda, N.; Katayama, K. Genogroup-Specific PCR Primers for Detection of Norwalk-like Viruses. *Journal of virological methods* **2002**, *100*, 107–114.
5. Park, J.-S.; Lee, S.-G.; Jin, J.-Y.; Cho, H.-G.; Jheong, W.-H.; Paik, S.-Y. Complete Nucleotide Sequence Analysis of the Norovirus GII.4 Sydney Variant in South Korea. *BioMed Research International* **2015**, *2015*, 1–7, doi:10.1155/2015/374637.
6. Trujillo, A.A.; McCaustland, K.A.; Zheng, D.-P.; Hadley, L.A.; Vaughn, G.; Adams, S.M.; Ando, T.; Glass, R.I.; Monroe, S.S. Use of TaqMan Real-Time Reverse Transcription-PCR for Rapid Detection, Quantification, and Typing of Norovirus. *Journal of Clinical Microbiology* **2006**, *44*, 1405–1412, doi:10.1128/JCM.44.4.1405-1412.2006.

7. Won, Y.-J.; Park, J.-W.; Han, S.; Cho, H.-G.; Kang, L.-H.; Lee, S.-G.; Ryu, S.-R.; Paik, S.-Y. Full-Genomic Analysis of a Human Norovirus Recombinant GII.12/13 Novel Strain Isolated from South Korea. *PLoS ONE* **2013**, *8*, e85063, doi:10.1371/journal.pone.0085063.
8. Lu, J.; Fang, L.; Sun, L.; Zeng, H.; Li, Y.; Zheng, H.; Wu, S.; Yang, F.; Song, T.; Lin, J.; et al. Association of GII.P16-GII.2 Recombinant Norovirus Strain with Increased Norovirus Outbreaks, Guangdong, China, 2016. *Emerging Infectious Diseases* **2017**, *23*, 1188–1190, doi:10.3201/eid2307.170333.
